# Silicone lymphadenopathy: A case report and literature review

**DOI:** 10.1097/MD.0000000000046777

**Published:** 2026-01-02

**Authors:** Xinyue Zhang, Siyi Luo, Qing Xiong, Zhichun Wang

**Affiliations:** a Department of Pathology, Jiujiang NO. 1 People’s Hospital, Jiujiang, People’s Republic of China; b Department of Breast, Jiujiang NO. 1 People’s Hospital, Jiujiang, People’s Republic of China.

**Keywords:** breast, case report, implantation of silicone gel prosthesis, silicone lymphadenopathy

## Abstract

**Rationale:**

Silicone Lymphadenopathy is a rare complication of breast silicone implantation. Our article reports a case of Silicone Lymphadenopathy and discusses related studies to enhance the accuracy of patient diagnosis and prevent misdiagnosis.

**Patient concerns:**

It has gradually occurred with the increase in follow-up cases of breast silicone implantation. Clinicians and pathologists should pay attention to the diagnosis of Silicone Lymphadenopathy.

**Diagnoses:**

The patient was diagnosed with Silicone Lymphadenopathy by hematoxylin-eosin staining and immunohistochemistry in pathology.

**Interventions:**

A biopsy tissue was performed on the patient’s lymph nodes. After pathological diagnosis, silicone lymphadenopathy was confirmed. No special treatment was given at that time.

**Outcomes:**

One year later, the patient showed no signs of discomfort.

**Lessons:**

As the number of breast augmentation patients increases, although silicone lymphadenopathy is a rare complication of breast augmentation, this condition is becoming more and more common. When diagnosing this disease, we need to thoroughly understand the patient’s medical history, including whether they have a history of breast augmentation or breast cancer, and pay attention to the differential diagnosis. Currently, there is no specific treatment method for this disease.

## 1. Introduction

At present, silicone is the most common material for breast filling, and its implantation in the body is closely related to the pathogenesis and development of silicone-induced lymph nodes.^[1]^ In the diagnosis of silicone-induced lymph nodes, computerized tomography and ultrasonography (US) can provide some diagnostic evidence, but in most cases, diagnosis under a pathological microscope is required.

## 2. Case presentation

**Figure 1.**
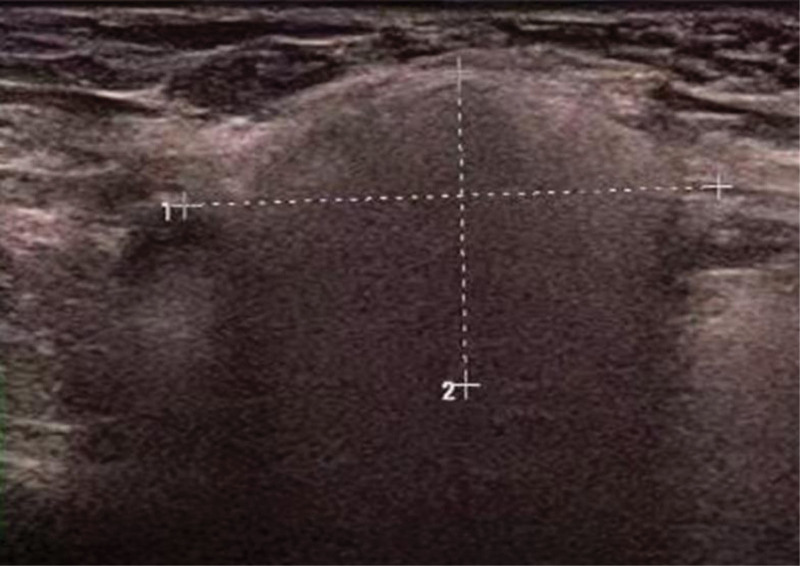
Several slightly strong echo masses can be seen in the right armpit, ~2 × 17 mm in size, with clear boundaries and uniform internal echoes.

**Figure 2.**
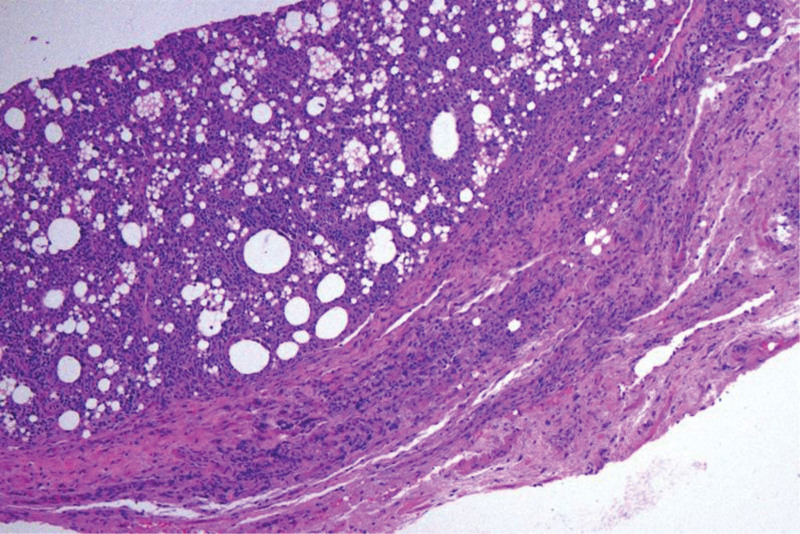
The microscopic expression of the biopsy tissue through HE staining shows the formation of vacuole-like substances and fibrous cystic substances (HE, ×100). HE = hematoxylin-eosin.

**Figure 3.**
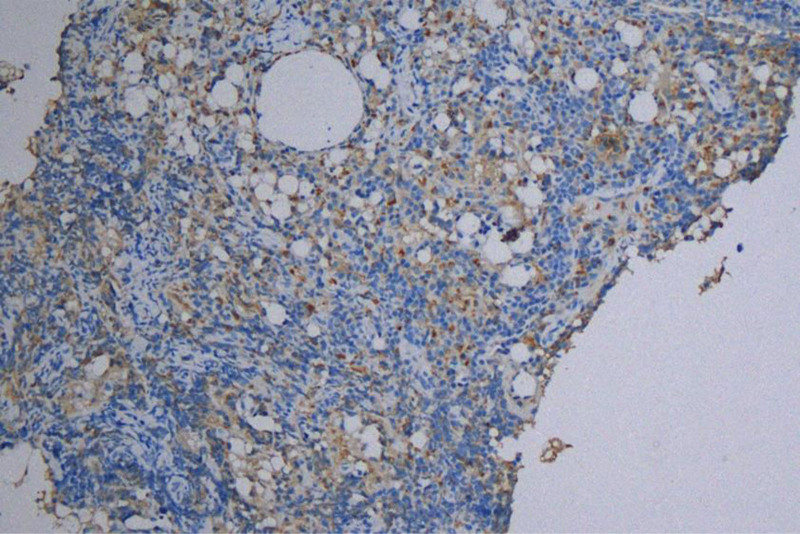
The IHC results showed that CD68 was positively expressed in the biopsy tissue (IHC, ×200). IHC = immunohistochemistry.

A 42-year-old female patient visited our hospital’s breast surgery department 3 years ago. She reported that she could feel multiple nodules in the right axilla, accompanied by tenderness. Our doctors recommended a US examination, which revealed abnormal echoes in the right supraclavicular fossa and subcutaneous tissue of the right axilla, suggesting extrusion of breast implants. After inquiring about the medical history, it was discovered that the patient had undergone bilateral breast implant surgery 10 years ago. At that time, our hospital did not provide any treatment measures for the patient. Last year, the patient returned for a follow-up medical examination, stating that she had removed the implants 1 year ago. Breast ultrasound showed several slightly hyperechoic masses in the right axilla, measuring ~28 mm × 17 mm, 30 mm × 19 mm, and 19.3 mm × 14.7 mm (Fig. 1). The boundaries were relatively clear, with uniform internal echoes and “snowstorm” appearance. Mammography showed patchy and nodular densities in the breast parenchyma, with uneven density increase, no definite masses or calcifications, normal skin and subcutaneous tissue, and a high-density nodule in the right axillary region, ~43 mm × 31 mm, seemingly composed of several fused nodules, with surrounding structures remaining intact. Multiple nodules were also found in the right supraclavicular and infraclavicular fossae, which may be enlarged lymph nodes. A puncture was performed on the right axillary nodule of the patient. After the biopsy tissue was sent to the pathology department, multiple vacuolated substances could be observed under the microscope, along with suspected fibrous capsule-like tissue (Fig. 2). The immunohistochemistry for CD68 was positive (Fig. 3). The final biopsy tissue diagnosis was silicone lymphadenopathy.

## 3. Discussion

Not only patients with damaged breasts choose silicone breast implants, but also ordinary people without any pain will choose silicone breast implants for aesthetic reasons. Silicone is a permanent, thermally stable, noncarcinogenic filler with stable viscoelasticity and minimal adhesion to surrounding tissues.^[2]^ Although the material properties indicate that the implant has no obvious adverse effects on the human body when placed inside, once the implant breaks and leaks, the consequences will still be detrimental to the human body. Complications may include wound rupture, hematoma formation, infection, and formation of foreign body granulomas.^[3]^ However, if the patient’s immune system is weak, pneumonia is more likely to occur. Currently, there have been reports of a certain connection between anaplastic large cell lymphoma and implants.^[2,4]^ The lymph node diseases in patients with silicone implants can be induced by granulomatous inflammation caused by gel migration, leading to cell-mediated immune responses and reactivation of T cell stimulation. Moreover, silicone particles can migrate to the local lymph nodes through macrophages in the reticuloendothelial system, resulting in swelling, fibrosis, and foreign body granuloma reactions.^[5,6]^ The lymphatic drainage of the breasts occurs through the axillary, thoracic, and internal mammary pathways in both forward and reverse directions.^[7]^ However, some literature indicates that the rupture of breast implants can lead to silicone tumors in multiple locations within the human body, suggesting that silicone leakage may be confined to the breast or spread to local lymph nodes, or even to distant organs, causing inflammation.^[8–11]^ It is difficult to confirm the rupture of silicone implants through physical examination methods.^[8]^

Silicone lymphadenopathy is a known rare complication of breast implant rupture. The axillary lymph nodes, internal mammary lymph nodes, and supraclavicular lymph nodes are most commonly affected, while distant lymph nodes such as mediastinum, thyroid, and lungs can also be affected.^[9,10,12–14]^ Silicone lymphadenopathy is often mistaken for a tumor in clinical diagnosis. Silicone leakage in breast prosthesis may cause local lymph node enlargement, and this situation may be similar to the manifestations of malignant tumors in imaging examinations.^[12,14]^ Although the typical “snowstorm” appearance manifestations on ultrasound are often regarded as the classic manifestations of silicone lymphadenopathy, with high sensitivity and specificity. However, studies have shown that the “snowstorm” appearance on US is related to the number of silicone-containing granulomas in the lymph nodes.^[15]^ When there are a small amount of silicone-containing granulomas, “snowstorm” appearance does not manifest.^[15]^ Although magnetic resonance imaging is regarded as the most standard imaging method for evaluating the rupture of silicone implants, the diagnosis of silicone lymphadenopathy by magnetic resonance imaging is obviously less definite than that by US.^[15,16]^ Since silicone lymphadenopathy may have fluorodeoxyglucose affinity, it is prone to be confused with malignant tumors when diagnosed by positron emission tomography–computed tomography.^[17]^ Although imaging diagnostics do have certain suggestive value in diagnosis, when it comes to the diagnostic role that requires significant guidance, advanced equipment and experienced radiologists are indispensable. Otherwise, it may lead to misdiagnosis and affect the communication between doctors and patients.^[18]^ Pathology is indispensable for the diagnosis of silicone lymphadenopathy. Currently, lymph node biopsy or lymph node resection biopsy is commonly used for diagnosis. Through hematoxylin-eosin staining, numerous cystic and vacuolar structures can be observed under the microscope. Surrounding these structures, there is tissue resembling the fibrous capsule wall. The foreign body giant cell reaction is usually mild. In individual cases, if there is a rupture outside the cyst, there can be more foreign body multinucleated giant cells, along with silicone, foam cells, and lymphocytes.^[19,20]^ CD68 serves as a marker for macrophages, indicating macrophage proliferation. Of course, in the diagnosis of silicone lymphadenopathy, in addition to the suggestive role of immunohistochemistry, it is also necessary to combine the microscopic manifestations of hematoxylin-eosin and relevant medical history. Attention should be paid to differentiating it from reactive lymphadenopathy, breast implant-related anaplastic large cell lymphoma, and other common breast lymphomas. In breast implant-associated anaplastic large cell lymphoma, most tumor cells have prominent nucleoli and the nuclei are round or oval. The chromatin of the cells is vesicular. In breast implant-related anaplastic large cell lymphoma, the tumor cell membranes strongly express CD30, and there is a significant increase in eosinophils. Reactive lymphadenopathy has normal lymph node structure and mixed cell types and does not have invasive growth or damage to the surrounding tissue structure. Diffuse large B-cell lymphoma is the most common type of lymphoma among primary tumor lymphomas of the breast.^[21]^ Under the microscope, medium to large cells can be seen diffused and growing invasively. Cytologic examination usually distinguishes between centroblastic, immunoblastic, and anaplastic subtypes.^[22]^ When the amount of tissue samples obtained through puncture or biopsy is insufficient, or when the samples do not show typical cellular or tissue structural changes, it is often difficult for pathologists to make an accurate diagnosis. At such times, the final diagnosis result may need to be combined with clinical and imaging-related data for a more comprehensive assessment.

Studies have shown that silicone lymphadenopathy often occurs 6 to 10 years after surgery, so knowing the patient’s history of breast implantation is particularly important for diagnosis.^[23]^ For breast cancer patients, clinicians should especially pay attention to differentiating this disease from metastatic breast cancer. When examining enlarged lymph nodes in women with a history of breast cancer, one should first rule out the rupture of breast implants and the resulting lymphadenopathy induced by silicone gel.^[24]^ It cannot be ignored that some patients also have the symptom of fever.^[25]^

In recent years, the number of people undergoing breast augmentation with implants has been increasing, and the number of patients with related complications has also been rising. However, silicone lymphadenopathy is also one of the rare complications associated with this procedure. Our article is a single-center case report and only provides relevant discussions on the diagnosis of the disease. However, the mechanism of the disease’s occurrence and development is still unclear, and more cases and related experiments are needed for fundamental prevention and treatment. The current view on the treatment of this disease is that no special treatment is necessary unless there are specific symptoms.^[26]^ We also conducted follow-up visits for the patient 1 year later, and the patient currently has no discomfort.

In conclusion, the research on the occurrence and development of silicone lymphadenopathy is still insufficient. The diagnosis of silicone lymphadenopathy requires a combination of the patient’s medical history, imaging examination results, and pathological examination results. Pathology, as one of the diagnostic methods, needs to pay attention to relevant differential diagnoses when diagnosing this disease, in order to improve the diagnostic accuracy.

## Author contributions

**Investigation:** Xinyue Zhang, Siyi Luo, Qing Xiong.

**Resources:** Zhichun Wang.

**Writing – original draft:** Xinyue Zhang.
